# Complete chloroplast sequence of *Fenerivia ghesquiereana* (Annonaceae): a rare and endemic tree from Madagascar

**DOI:** 10.1080/23802359.2020.1855604

**Published:** 2021-01-16

**Authors:** Yixi Wang, Tijana Cvetković, Amy N. A. Aritsara, Angelo D. A. Carrion, Joeri S. Strijk

**Affiliations:** a Biodiversity Genomics Team, Guangxi Key Laboratory of Forest Ecology and Conservation, Guangxi University, Nanning, China; b Plant Ecophysiology and Evolution Group, College of Forestry, Guangxi University, Nanning, China; c Département Forestière et Environnement, Ecole Supérieure des Sciences Agronomiques, Université d’Antananarivo, Antananarivo, Madagascar; d Pha Tad Ke Botanical Garden, Alliance for Conservation Tree Genomics, Luang Prabang, Laos

**Keywords:** *Fenerivia ghesquieriana*, complete chloroplast genome, Annonaceae, Malmeoideae, phylogenomics

## Abstract

*Fenerivia ghesquiereana* (Cavaco & Keraudren) R.M.K. Saunders (Annaonaceae) is a rare and endemic tree restricted to the warm tropical forests of Madagascar, a major global hotspot for biodiversity. Species in the genus are mostly restricted to a thin belt along the eastern edge of the island and remain under intense pressure due to deforestation for logging, mining and slash-and-burn agriculture (‘*tavy*’), despite national pledges to curb biodiversity losses and increase park protection to combat illegal logging and poaching. Here we report the complete chloroplast genome sequence of this species to support ongoing efforts to complete the (sub-)tribal classification of the family. The chloroplast sequence of *F. ghesquiereana* was 160,194 bp in length, including two inverted repeat regions of 26,093 bp, a large single-copy region of 89,041 bp and a small single-copy region of 18,967 bp. A total of 160 genes were annotated, of which 115 are coding, 37 are tRNA genes, and eight are rRNA genes. The overall GC content was 39%; this was higher in the IRs (43.4%) when compared to the LSC (30.7%) and the SSC (33.9%) regions. A Maximum Likelihood phylogenetic analysis with a selection of other plastomes in Annonaceae placed *F. ghesquiereana* as sister to *Meiogyne hainanensis* (Merr.) in subfamily Malmeoideae.

The Annonaceae are a diverse family of ca. 108 genera and 2400 species of trees, shrubs and lianas (Chatrou, Pirie, et al. 2012). They are among the most important components of tropical forests worldwide (Couvreur et al. 2018). Many species are also widely cultivated throughout the tropics and subtropics for their fleshy edible fruits, and for their spice, aromatic, and medicinal properties (Chatrou, Erkens, et al. 2012; Strijk et al. 2020).

The genus *Fenerivia* (∼10 spp.) is endemic to Madagascar (Saunders et al. 2011) and was included in the genus *Polyalthia* until recently. Morphological and phylogenetic studies (based on nuclear and chloroplast markers) have provided support for its recognition as an independent genus embedded in tribe Fenerivieae of the Malmeoideae subfamily (Deroin 2007; Saunders et al. 2011; Chatrou, Pirie, et al. 2012).

*Fenerivia ghesquiereana* (Cavaco & Keraudren) R.M.K. Saunders (vern. name ‘*Korofoka*’) is a rare tree confined to the wet tropical rain forests lining the eastern edge of Madagascar. Here, we report the complete chloroplast sequence of *F. ghesquiereana* (Cavaco & Keraudren) R.M.K. Saunders. To date, plastome data for the genus was not available and could not be included in any major molecular workup of the family. This new data will contribute to the delineation of relationships of *Fenerivia* with other genera in future studies.

We extracted genomic DNA from silica-dried leaves collected separately Madagascar (voucher ARI31, 14°40′48.5″S, 49°31′43.1″E) deposited in the TEF herbarium at Antananarivo, using a modified SDS protocol (Hinsinger & Strijk 2017). Library construction and sequencing were performed by Novogene (Beijing, China), using an Illumina HiSeq2500 platform following system manufacturer instructions. We conducted a *de novo* assembly of the chloroplast genome with Novoplasty v3.8.2 (Dierckxsens et al. 2017) using one GB of sequence data. The annotation was performed with GeSeq (Tillich et al. 2017) and was manually adjusted in Geneious (R9, Biomatters, www.geneious.com).

In *F. ghesquiereana*, the complete chloroplast genome (GenBank accession MT780300) was 160,194 bp in length, including two inverted repeat (IRS) regions of 26,093 bp, a large single-copy (LSC) region of 89,041 bp and a small single-copy (SSC) region of 18,967 bp. 160 genes were annotated, of which 115 were coding, 37 were tRNA genes, and eight were rRNA genes. The overall GC content was 39%. GC content was higher in the IRs (43.4%) than in LSC (30.7%) and SSC (33.9%) regions.

We explored phylogenetic relationships of *F. ghesquiereana* with other species in Annonaceae and outgroup species in Myristicaceae and Magnoliaceae, using additional plastomes available in GenBank (Annonaceae: *Annona cherimola* Mill. NC030166; *Meiogyne hainanensis* (Merr.) Tien Ban NC043867; *Uvaria macrophylla* Roxb. NC041442. Magnoliaceae: *Magnolia decidua* (Q.Y. Zheng) V.S. Kumar MK934524; *Liriodendron tulipifera* L. MK477550. Myristicaceae: *Knema elegans* Warb. MK285564; *Myristica yunnanensis* Y.H. Li MK285565).

The four main chloroplast regions (LSC, IRb, SSC and IRa) were automatically extracted, re-positioned and aligned using the software EcuADOR (Armijos Carrion et al. 2020) for all new and downloaded sequence data. A Maximum Likelihood phylogenetic tree was reconstructed using RaxML-NG (Kozlov et al. 2019) using 1000 bootstrap replicates. *Fenerivia ghesquiereana* was placed as sister to *Meiogyne hainanensis* in subfamily Malmeoideae with species in the Annonoideae subfamily as sister (*Annona cherimola* and *Uvaria macrophylla*) (Figure 1).

**Figure 1.**
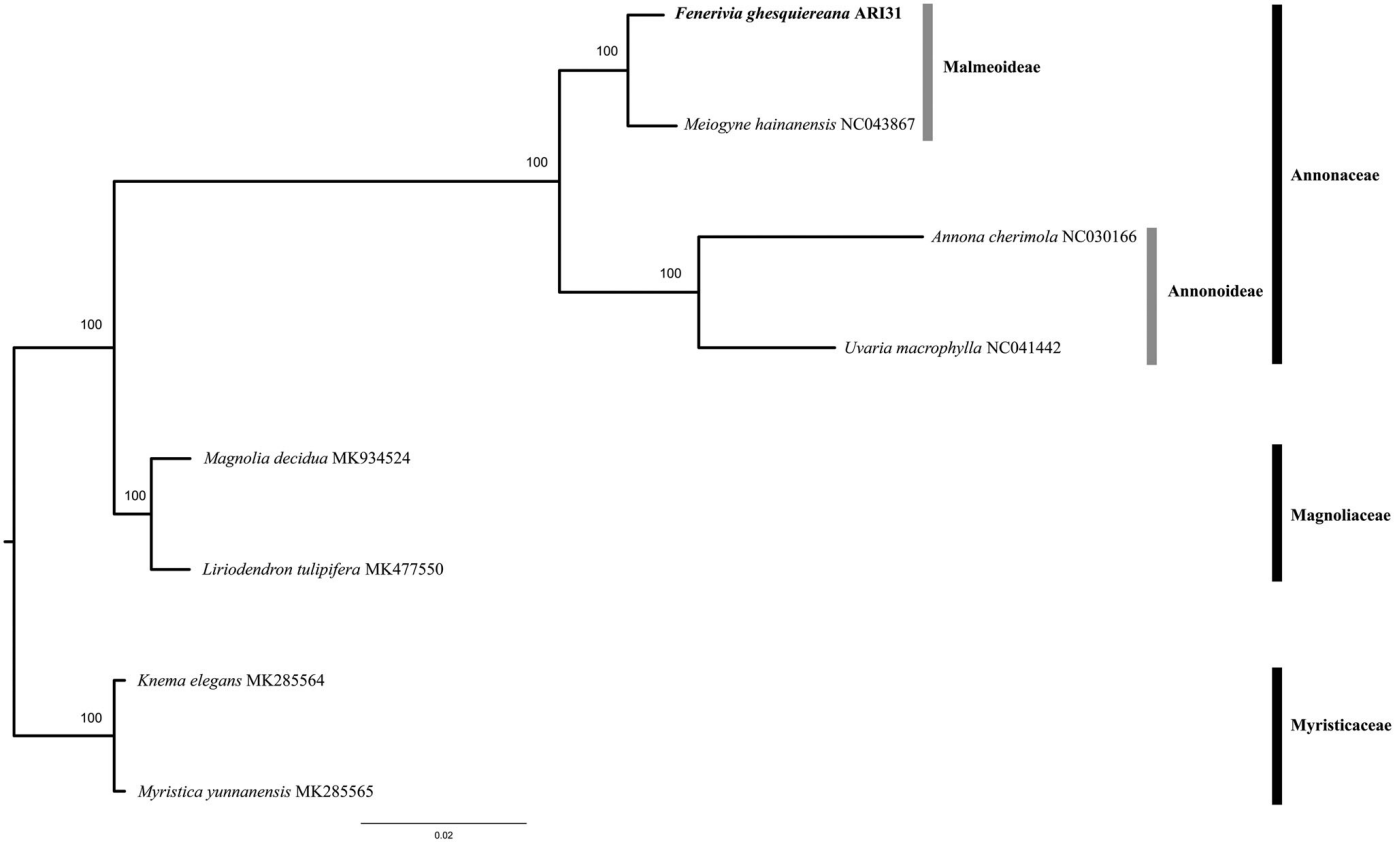
Maximum Likelihood tree using full plastome data of *Fenerivia ghesquieriana*, *analyzed* together with other Annonaceae and outgroup species retrieved from GenBank. Numbers above branches indicate ML bootstrap values.

## Data Availability

The data that support the findings of this study are available in GenBank (https://www.ncbi.nlm.nih.gov/) under accession number MT780300.
